# The Histological Background of Recurrence in Laryngeal Squamous Cell Carcinoma: An Insight into the Modifications of Tumor Microenvironment

**DOI:** 10.3390/cancers15123259

**Published:** 2023-06-20

**Authors:** Giorgia Arcovito, Annarita Palomba, Oreste Gallo, Alessandro Franchi

**Affiliations:** 1 Section of Pathology, Department of Translational Research, University of Pisa, 56126 Pisa, Italy; g.arcovito@studenti.unipi.it; 2 Unit of Histopathology and Molecular Diagnostic, Azienda Ospedaliera Universitaria Careggi, 50139 Florence, Italy; annarita.palomba@aouc.unifi.it; 3 Department of Experimental and Clinical Medicine, University of Florence, 50139 Florence, Italy; oreste.gallo@unifi.it

**Keywords:** laryngeal carcinoma, recurrence, radiotherapy, surgery, microenvironment

## Abstract

**Simple Summary:**

Laryngeal cancer is a relatively common neoplasm of head and neck, whose management hinges on a combination of treatments such as surgery, radiotherapy and chemotherapy. Tumor recurrences may present important differences from the primary tumor that largely depend on previous treatments. the immune system plays a crucial role in the natural course of the disease, owing the capability to influence its behavior through a complex interaction of mechanisms. For this reason, the use of immunotherapy in addition to standard therapies is increasingly gaining importance nowadays and the selection of patients who can benefit the most from this treatment can help optimizing its success. However, conventional treatments can induce relevant changes in the host immune response, thus affecting tumor progression and patient outcome. This fact should be taken into account when planning immune-based treatments like immune checkpoint inhibitors. In this article, we review the histologic and molecular treatment-induced changes that may affect the diagnosis of recurrent laryngeal cancer, the assessment of predictive markers, and the response to treatment with immune checkpoint inhibitors.

**Abstract:**

Recurrent laryngeal carcinoma presents differences from the primary tumor that largely depend on the treatment. In this article, we review the histologic and molecular treatment-induced changes that may affect the diagnosis of recurrent laryngeal carcinoma, the assessment of predictive markers, and the response to treatment with immune checkpoint inhibitors. Radiotherapy induces profound modifications that are strictly related to necrosis of different tissue components, fibrosis, and damage of the tumor vessels. Postradiotherapy recurrent/persistent laryngeal squamous cell carcinoma typically presents a discohesive growth pattern within a fibrotic background associated with significant changes of the tumor immune microenvironment, with both important immunosuppressive and immunostimulatory effects. Overall, the increase of immunoregulatory cells and immune checkpoints such as CTLA-4, TIM-3, PD-1, and PD-L1 induced by radiotherapy and chemotherapy strongly supports the use of immune checkpoint inhibitors in recurrent/persistent laryngeal carcinoma. Future studies aiming to identify predictive factors of the response to immune checkpoint inhibitors should consider such treatment-induced modifications.

## 1. Introduction

Laryngeal cancer is the second most common cancer of the upper aerodigestive tract, accounting for approximately 20% of all head and neck malignancies [1]. The age standardized incidence rates in Europe for the year 2020 were 9.8 × 100,000 and 1.4 × 100,000, while mortality rates were 4.8 × 100,000 and 0.5 × 100,000 for males and females, respectively [2]. High tobacco exposure and alcohol consumption are the two major well-established risk factors, whereas HPV infection seems to play a marginal role at this anatomic site [3]. Laryngeal cancer is treated using a combination of chemotherapy, radiation, and surgical techniques, depending on the histologic type, biology, location, and stage, as well as patient and other factors. Early glottic cancer can be treated either with curative radiotherapy (RT) or with surgery, ensuring a comparable overall survival. RT is often preferred as primary treatment because it grants a better functional integrity of the larynx, and the risk of local recurrence is comparable with that of surgical treatments [4]. Treatment for locally advanced T3 and T4 laryngeal cancers includes total laryngectomy with or without postoperative RT, but since the introduction of organ preservation techniques, including RT with or without chemotherapy, the number of total laryngectomies performed has drastically reduced [5]. However, the incidence of local recurrence can be as high as 35–50% [6,7], and salvage surgery is generally performed in these cases.

In recent years, the tumor immune microenvironment has been the object of extensive studies, and as a result, immune check point inhibitors (CPI) have been introduced in the treatment of recurrent/metastatic head and neck squamous carcinoma as valid alternative to standard chemotherapy. In general, the clinical benefits of these treatments have been quite variable, hence the need to select those patients who may obtain the maximal efficacy through the identification of predictive biomarkers. Moreover, among the critical issues that must be considered in the evaluation of the efficacy of CPI as well as in the assessment of predictive biomarkers are the changes in the tumor microenvironment that occur after primary treatment in recurrent/persistent SCCs. In this review, we focus on the changes induced by treatment on laryngeal SCC, with an emphasis on the molecular mechanisms involved in the modifications of tumor microenvironment in recurrent tumors and a correlation with their histopathologic features.

## 2. Histopathologic Changes of Laryngeal Anatomic Structures after Radiotherapy

Among the several anatomic components, the most relevant changes induced by radiotherapy involve the vasculature [8]. The early effects on blood vessels include detachment of endothelial cells from the basement membrane and apoptosis [9], and this damage is greater in small capillaries of the tumor bed because their wall lacks a pericytic layer and it is mainly formed by the endothelium [9]. These vessels are often dilated and may be occluded by thrombosis due to the prothrombotic state created by radiation and the endothelial damage [9]. Small- and medium- sized arteries develop subendothelial or adventitial fibrosis, hyalinization of the media, and accumulation of lipid-laden macrophages in the intima, a picture that is virtually indistinguishable from atherosclerosis. Overall, this vascular damage results in hypoxia of the tumor microenvironment. Accordingly, radiation-induced microvascular damage in the irradiated larynx includes the presence of telangiectatic capillaries as well as the thickening and hyalinization of the arteriolar wall [10]. 

A constant delayed tissue change of radiotherapy is subepithelial fibrosis, which consists of areas of dense acellular or paucicellular collagen with variable extension and severity [11]. Laryngeal mucosa is expanded by dense collagen deposition, and fibrin may also be detected in the stroma in between collagen fibers and fibroblasts [10]. In their study of 20 irradiated vocal folds from 13 patients, Berg et al. found increased collagen and muscle fiber disorganization in the irradiated specimens as compared to the controls, together with a significant increase of hyaluronic acid and fibronectin tissue content, and a decrease of laminin [12]. This is accompanied by increased transcription of markers for fibrosis, oxidative stress, inflammation, glycosaminoglycan production, and apoptosis [13]. The mechanisms involved in the development of vocal fold fibrosis have been investigated in murine models [13,14], which show similar histologic and biochemical changes to irradiated human vocal folds. Transcriptional analysis revealed upregulated expressions of TGF-beta1, which is responsible for the fibrotic changes and induces myofibroblast differentiation, and iNOS at six months. Conversely, the expressions of Acta2, Col1a1, Col3a1, and MMP8 were downregulated, indicating reduced collagen turnover [14]. Another characteristic radiotherapy-induced delayed mesenchymal change in the upper respiratory tract is the presence of atypical fibroblasts, which can be detected in 50–60% of the cases in salvage laryngectomies [10]. They present as large cells with amphophilic/basophilic cytoplasms and large irregularly hyperchromatic nuclei with prominent nucleoli.

Both the lining epithelium and salivary-type glands of the laryngeal mucosa are affected by radiation injury. In the acute phase, necrosis of the epithelium predominates, although ischemia resulting from vascular damage may also cause delayed necrosis [11]. Epithelial atrophy is a delayed effect, and histologically consists of thinning of the surface epithelium, loss of salivary gland-type acini, and sialometaplasia (squamous metaplasia) of the acini and ducts in the remaining glands. Importantly, as a delayed effect, epithelial cells may present cellular atypia with enlargement of both nucleus and cytoplasm, but these changes are not considered premalignant [11]. However, more complex architectural alterations may develop as well, resulting in true dysplastic changes with potential malignant evolution [11,15].

## 3. Recurrent Squamous Cell Carcinoma: Histopathologic Changes in Postradiotherapy Recurrence vs. Postsurgical Recurrence

The changes induced by radiation in normal tissues can be detected in specimens from salvage surgery or after recurrence of head and neck squamous cell carcinoma (HNSCC) as well. However, they combine with important modifications induced by the tumor itself. Unfortunately, systematic histopathologic studies describing the tumor changes in persistent/recurrent laryngeal SCC after radiotherapy or chemotherapy in comparison with persistent/recurrent SCC after conservative surgeries are lacking. Pandya et al. examined 27 cases of oral SCC that recurred after radiation therapy within an average span of 11 months and compared their histologic features with those of 26 non-irradiated cases of oral SCC [16]. As expected, irradiated carcinomas presented significantly increased fibrinous exudates, necrosis, and vessel wall thickening in comparison with nonirradiated cases [17]. Moreover, intrinsic changes in the histologic features of the irradiated tumors included a decrease of the degree of keratinisation and inflammation, whereas nuclear pleomorphism was significantly increased [16]. Interestingly, a significant modification of the pattern of invasion was observed in the irradiated carcinomas, consisting mainly of small groups of tumor cells forming strands and cords within the dense collagenous stroma. Altogether, these modifications of the histologic tumor features seem to point towards a loss of differentiation and an increase of aggressiveness, but this still needs confirmation. Noteworthily, radiation exposure has been proved to promote epithelial-mesenchymal transition (EMT) in tumor cells, a biologic process that is responsible for a loss of intercellular contacts and the acquisition of a mesenchymal phenotype, implying migration properties and a tendency toward invasion and dissemination [17]. Neoplastic cells undergoing EMT are in turn capable of remodelling extracellular matrices through the secretion of proteases, favouring invasiveness and metastatic spread [18,19]. The main radio-mediated mechanism inducing EMT involves the activation of fibroblast in cancer-activated fibroblast (CAFs), which produces an array of cytokines and growth factors after irradiation promoting EMT, primarily represented by TGF-beta and (C-X-C motif) ligand 12 (CXCL12)/stromal derived factor1 (SDF-1) [18,20,21]. In addition, radiotherapy increases EMT through the recruitment of tumor-associated macrophages (TAMs) producing TGF-beta and the increase of reactive oxygen species (ROS) activating several pathways such as Wnt, TGF-b, NF-kB, Notch, and HIF-1 [17]. Such RT-mediated modifications occurring in extracellular matrices could possibly explain the histological appearance with loss of cohesiveness at the leading edge of the tumor that is typically observed after radiotherapy [22].

**Figure 1:**
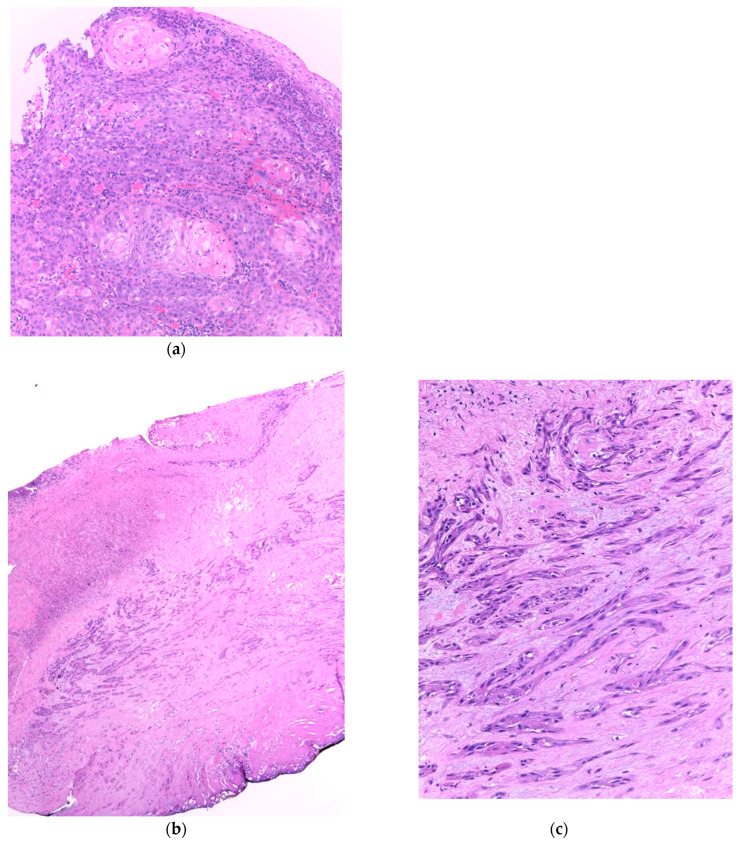
(**a**). Incisional biopsy of a moderately differentiated squamous cell carcinoma of the vocal cord. The tumor consists of islands of atypical squamous cells with areas of keratinization. There is a light inflammatory infiltrate beneath the surface epithelium. (**b**) Recurrent squamous cell carcinoma of the vocal cord following radiotherapy. (**c**) The surface epithelium is attenuated or absent and there is fibrosis of the superficial mucosa. The tumor is visible in the mid part of the section and consists of elongated nests and small groups of neoplastic cells separated by fibrotic stroma.

**Figure 2:**
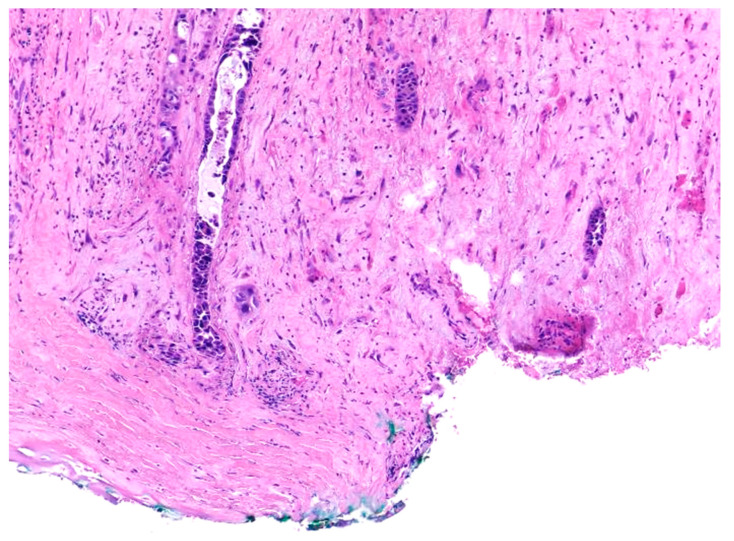
Recurrent squamous cell carcinoma of the vocal cord following radiotherapy. The histologic section shows the deep margin of a specimen of vocal cord resection, inked in green. The tumor consists of small, separated nests of tumor cells and it is thus impossible to assess the margin status with certainty.

**Figure 3:**
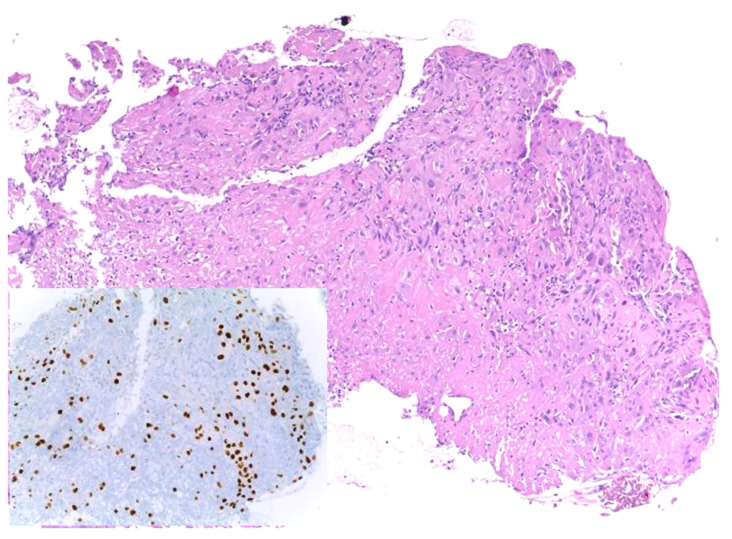
Recurrent squamous cell carcinoma of the vocal cord following radiotherapy. In this biopsy specimen, tumor cells are obscured by necrosis and fibrosis. They are highlighted by the nuclear immunohistochemical staining for P40 (inset).

**Figure 4:**
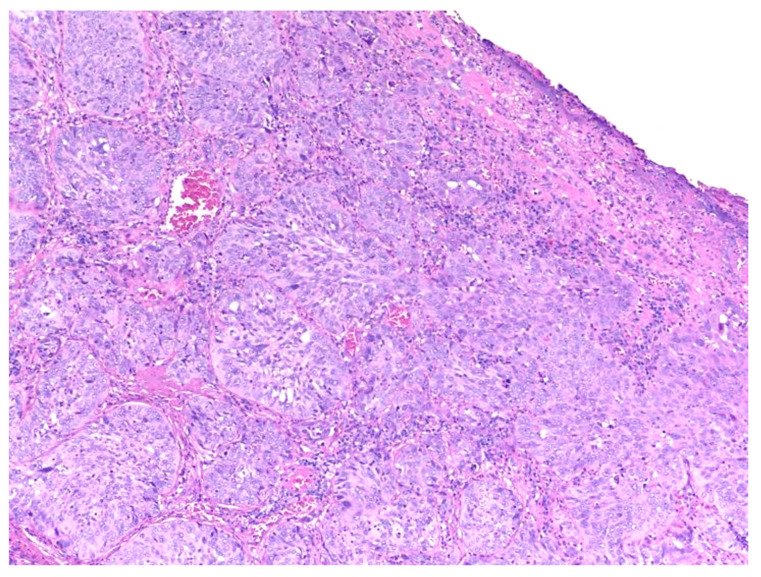
Recurrent laryngeal squamous cell carcinoma following surgery. The tumor presents with cohesive irregular nests of neoplastic cells.

Similar changes can be observed in recurrent/persistent postradiation laryngeal SCCs. Figure 1 illustrates a postradiation recurrent SCC of the vocal cord. The tumor shows no connection with the surface epithelium and consists of cords and a small island of tumor cells within a fibrotic stroma. Although the significance of these findings for the biology of the tumor remains to be fully determined, they nevertheless have an impact on the evaluation of resection margins (Figure 2), as well as on the interpretation of histopathologic findings in biopsies, where neoplastic cells may be difficult to identify in small superficial samples, or if they are set within necrosis or fibrosis (Figure 3). In comparison, postsurgical recurrences of laryngeal SCC consist of irregular infiltrative tumor islands, but necrosis, acellular fibrosis with collagenization, inflammation, and the characteristic damage to vessels are usually absent (Figure 4).

## 4. Treatment-Induced Modifications of the Tumor Microenvironment

**Figure 5:**
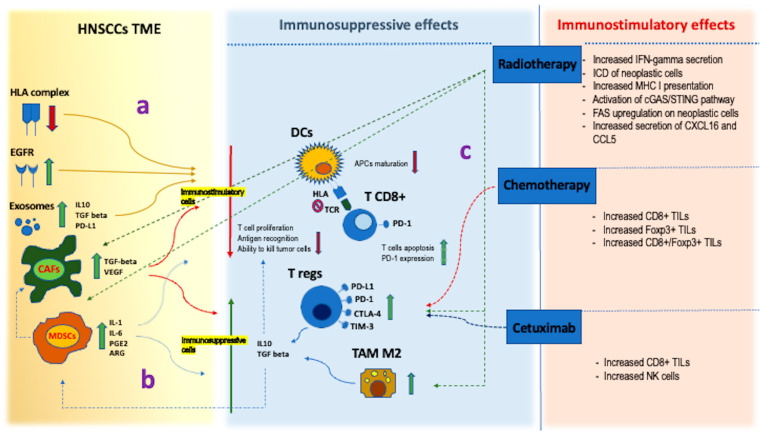
The degree of immunosuppression in posttreatment laryngeal SCC TME relies upon several factors fostering each other. SCC-related alterations of TME are responsible for a reduction of immune response by decreasing the activity of immune-stimulatory cells. Downregulation of the HLA complex impairs the process of antigen recognition by CD8+ T-cells [25]. Increase of tumor-derived exosomes alters TME toward an immunosuppressive fashion environment in many ways, including the inhibition of T-cells function and the modification of many several immunoregulatory factors such as PD-L1, IL-10, and TGF-beta [26]. EGFR is upregulated in most HNSCC, and activation of its downstream pathway hampers immune activation through several mechanisms, including downregulation of antigen presentation [27] (**a**). MDSCs and CAFs are typically increased in HNSCC TME, and they secrete a number of cytokines and soluble factors suppressing CD8+ T-cells, such as prostaglandins (PGE2), arginase (ARG), IL-1, IL-6, TGF-beta, and VEGF. MDSCs and CAFs exert immune suppressive roles both by decreasing the activity of immunostimulatory cells and recruiting immunosuppressive cells represented by Tregs and TAM M2. The latter produce immunosuppressive cytokines such as IL-10 and TGF-beta, which in turn further increase MDSCs within the TME and suppress T-cells functions [28,29,30,31,32] (**b**) T-regs are remarkably increased in TME of HNSCC and they show an upregulation of immune checkpoint molecules, namely PD-1, PD-L1, and CTLA-4 [33,34,35]. PD-1 expression is also increased on T CD8+ cells in HNSCCs [29,30]. On the other side, RT, CT, and Cetuximab are capable of both immunosuppressive and immunostimulatory effects. Radiotherapy contributes to the increase of MDSCs and CAFs, thus indirectly enforcing the immunosuppressive mechanisms described above [28]. At the same time, RT, CT, and Cetuximab increase Tregs in TME and favour the upregulation of PD-L1, PD-1, CTLA-4, and TIM-3 on the Tregs surface. Furthermore, RT recruits TAM M2, which, as already pointed out, fulfils an immunosuppressive activity [28] (**c**).

The TME encompasses a dynamic ensemble of elements comprising immune cells, nonimmune cells, and extracellular components, taking active part in the milieu where neoplastic cells develop [23]. Cells involved in TME accomplish a dual role in cancer imbalance, simultaneously favoring and hampering its progression in a tricky biological dialogue [19]. The immune cells include myeloid derived stem cells (MDSCs), regulatory T-cells (T-regs), tumor-infiltrating lymphocytes (TILs), TAMs, and dendritic cells (DCs). Nonimmune cells are mainly represented by CAFs. TME balance hinges on a constant interplay between immune cells, CAFs, and neoplastic cells which is modulated by several factors such as cytokines, chemokines, growth factors, extracellular matrix, and exosomes [24]. A tumor can be regarded as a complex ecosystem in which neoplastic cells interact with TME components, undergoing a process of coevolution in which they acquire a temporally and spatially heterogeneous phenotype with selective advantages such as the capability for immune evasion [19]. Furthermore, in parallel with the changes induced in the different histologic components, treatments also deeply modify the interactions between the several elements of the tumor microenvironment, ultimately affecting the growth of persistent/recurrent tumors (Figure 5). Most studies concerning TME modifications in recurrences of head and neck squamous cell carcinoma (HNSCC) have considered different anatomic sites altogether, mainly focusing on the alterations induced by radio and chemotherapy. The results of these studies are summarized in Table 1, which details the percent of laryngeal SCCs included in each study.

TME of laryngeal SCC has shown evidence of peculiar features which are responsible for a significant decrease in antitumoral response compared with SCCs of other districts [28,29,30,31]. The immunosuppressive environment of laryngeal cancer could be partly attributed to selective events following genetic alterations induced by alcohol and tobacco consumption [29]. Indeed, tobacco smoking has shown a meaningful association with immunosuppression and lowering of cytotoxic activity in TME [33]. Suppression of immune activity in HNSCC TME takes place through several mechanisms, including downregulation of HLA, which impairs neoplastic cells recognition by T-cells [25], upregulation of epidermal growth factor (EGFR) with impairment of an efficient immune activation [27], and production of tumor-derived exosomes inducing CD8+ T-cells apoptosis [26,28,29,30,31]. Moreover, antitumoral response is weakened by CAFs, which represent a relevant component of HNSCCs TME, especially in advanced-stage disease [32]. CAFs release transforming growth factor (TGF) beta and vascular endothelial growth factor (VEGF), inhibiting T-cells proliferation and promoting T-regs development. They also foster immune escape by recruiting TAM M2 subtypes which fulfill an immunosuppressive effect. Finally, immune modulation is further supported by MDSCs secretion of IL-1 and IL-6, which impairs APCs maturation and favors T-regs development [28,29,30,31]. T-regs increase has been proved both in TILs and circulating T-cells of HNSCC compared with healthy donors and has been associated with the upregulation of immune checkpoint molecules such as PD1, PDL1, and CTLA4 [33,34,35]. Therefore, the presence of an immunosuppressive microenvironment in HNSCC represents a critical issue to overcome. In this respect, the rationale of an immunotherapy based on the inhibition of the immune escape appears to be a reasonable strategy, especially when combined with chemo- and radiotherapy [44,45].

### 4.1. Radiotherapy Effects on TME

Radiotherapy can profoundly affect TME of laryngeal cancer, exerting both immunosuppressive and immunostimulatory effects [28,29,30,31,46]. Radiation exposure has been traditionally associated with immunosuppressive effects, due to the high susceptibility of immune cells to ionizing radiations, despite some differences among lymphocytic subsets in their radiosensitivities. Indeed, circulating CD8+ T-cells seem to be the most radiosensitive, while preexistent intratumoral T-cells are more radioresistant and are meant to perform the antitumoral effect [28]. Of note, T-regs hold a marked radioresistance, fairly contributing to the suppression of immune response following radiations [47]. However, radiotherapy enhances immune suppression in TME also by the recruitment of immunoregulatory cells, namely TAM M2, T-regs, and MDSCs, whose immunoregulatory functions undergo further modulation due to radiation effects [8,28]. For instance, T-regs show an upregulation of CTLA-4, which in turn inhibits T-cells functions [8]. Since TME of HNSCC is particularly enriched with immunosuppressive elements, one can assume that immunosuppression in the TME of laryngeal SCC gets strengthened after radiotherapy. It is important to underline that radiotherapy deeply influences laryngeal SCC TME toward an immunostimulatory trend as well, through the recruitment and activation of the host immune response against tumors [28,48]. Among all TME cellular elements, intratumoral T-cells proved to be the main actors involved in radiation-induced antitumoral activity, and despite undergoing a diminished proliferative capacity, they showed an increased production of IFN gamma after RT, which is crucial for tumor control [47].

Mechanisms contributing to this complex process are numerous and involve different pathways [28,46]. Firstly, radiation induces the immunogenic cell death (ICD) of cancer cells, which is a recently described cell death modality responsible for the activation of host immune response against dying cells antigens [31,47,49]. In detail, radiations induce ICD by killing neoplastic cells which subsequently release the danger-associated molecular patterns (DAMPs), the latter eliciting an antigen-specific immune response. DAMPs include several types of molecules such as calreticulin, which is translocated from the endoplasmic reticulum to the cell surface and represents an ‘eat me’ signal for the DCs. Among others, they also include HMGB1 and ATP, which are released in the extracellular milieu. All these molecules account for danger signals, in turn favoring DCs-associated cross-priming of CD8+ CTLs [28,46,48,49,50]. Once activated, CD8+ T-cells target neoplastic cells both inside the irradiated field and at distant sites from the neoplastic burden, configuring a peculiar immune-mediated phenomenon that has been named the abscopal effect [51,52,53]. Other mechanisms contributing to the abscopal effect have been investigated in some in vitro and in vivo experiments on various tumor cell lines. For example, the increase of MHC class I presentation occurs as a direct effect of ionizing radiations in a dose-dependent manner, which could enhance antitumoral CTLs response [54,55]. Considering these findings, some studies aimed to verify the clinical impact of the abscopal effect, especially questioning a hypothetic enhancement coming from the association between RT and ICIs. Evidence of a true clinical benefit is lacking, and results emerging from clinical studies in HNSCCs did not show any significant responses in nonirradiated lesions of patients treated with RT [56]). Radiation-induced cell damage also contributes to the extracellular release of cytosolic DNA which triggers the activation of the cGAS/STING pathway, upregulating type-I IFN signaling; IFN beta production is paramount for improving DCs activation and subsequent T-cells cross-priming [28,46,57,58]. In addition, radiation can also induce a durable upregulation of FAS (CD95) on neoplastic cells, which may allow their destruction by activated CTLs via Fas-dependent mechanisms [55,59,60]. Finally, radiotherapy can directly increase the secretion of CXCL16 and CCL5, which exert proinflammatory effects with the recruitment of immune cells [48].

Besides the immune compartment, stromal cells have also shown susceptibility to radiations, being influenced by the overall radiation-induced increase of proinflammatory cytokines such as IL-1 beta, IL-6, IL-8, and, most importantly, TGF-beta [8,46]. The mainly affected stromal elements of TME are CAFs. They showed loss of mobility and tumor-invasive capability along with prolonged survival, potentially due to the enhancement of focal contacts mediated by integrins alfa2, alfa5, and beta1, whose expression turned out to be increased by radiation [8]. CAFs are responsible for several immunosuppressive effects on TME, and have been linked to higher clinical stages and local recurrences in HNSCCs [8,32]. Besides, they interact with tumor cells by the secretion of cytokines, chemokines, and exosomes which contribute to ECM remodeling [19]. Particularly, their density is positively correlated with tumor spread through hematogenous, lymphatic, and perineural invasion [32]. Thus, it could be argued that the stabilization of CAFs induced by radiotherapy worsens the prognoses of patients with HNSCCs. The effects of radiotherapy on CAFs still need full clarification [8].

### 4.2. Chemotherapy and Cetuximab Effects on the TME

Along with radiation-induced modifications of TME in HNSCCs recurrences, chemotherapy’s effects on the antitumoral immune response are worth mentioning as well. In advanced HNSCCs, it has been demonstrated that TPF (docetaxel, cisplatin, and fluorouracil) induction chemotherapy increases TILs CD8+ and Foxp3+, which is a marker of T-regs. In particular, CD8+ TILs showed an increased density after chemotherapy, and Foxp3+ TILs underwent an increasing, yet not statistically significant, trend. Noteworthily, there has also been observed a higher CD8+/Foxp3+ TILs ratio after treatment, which is predictive of a good prognosis and better response to immune checkpoint inhibitors [36]. To this respect, it has been demonstrated that high CD8+ TILs infiltration is significantly associated with better outcome in terms of disease-free survival (DFS) and tumor relapses in advanced laryngeal cancers treated with definitive chemoradiation [61,62]. Conversely, another study carried out on post-CTRT HNSCC recurrences, including mainly laryngeal cancers, failed to demonstrate any changes in immune parameters concerning TILs density, HLA class I expression, and programmed death-ligand (PD-L) 1/2 upregulation on immune and neoplastic cells [37]. Lastly, cetuximab has been proven to induce NK cells activation in the TME of HNSCC, which in turn produces IFN gamma improving DCs maturation [63]. On the other hand, it causes an increase of Foxp3+ and CTLA4+ T-regs, whose activity inhibits NK cells cytotoxicity fostered by treatment itself [38]. Interestingly, HNSCC TME is distinctly enriched with NK cells, even when compared with other highly immune infiltrated cancer types [28]. Moreover, cetuximab enhances CD8+ T-cells density in HNSCCs TME, including PD-1+ and TIM3+ subsets, which exert an immune suppressive function. The upregulation of immune checkpoint receptors after cetuximab is predictive of worse prognosis and provides a further reason to add immune checkpoint inhibitors to cetuximab, in order to optimize immune-activating effects of the latter [39].

## 5. Modifications of PDL1 Expression in Recurrences

Programmed death-ligand 1 (PD-L1), also known as B7-H1, is a transmembrane protein belonging to the B7 superfamily, and it is mainly expressed on neoplastic cells and mononuclear immune cells. It binds to the PD-1 receptor expressed on T-cells, activating an inhibitory pathway that leads to their anergy, thus inducing a suppression of the antitumoral immune response [64]. It has been found in the tumor microenvironment of several solid cancers, including a significant subset of HNSCCs, where it has been detected both on tumor cells and tumor infiltrating mononuclear cells (TIMCs) [65]. Following the approval of immune checkpoint inhibitors pembrolizumab and nivolumab by FDA and EMA in the treatment of recurrent and metastatic HNSCCs, PD-L1 evaluation gained a pivotal importance in the diagnostic algorithm of HNSCCs, but its assessment still suffers from a lack of solid standardization [66,67]. This weakness is due to several reasons, including intratumoral heterogeneity, modifications occurring along the course of the disease, differences among the cutoffs, and exogenous modifications induced by local and systemic therapies [40,67,68].

Considering the effects of chemo/radiotherapy on PD-L1 expression in HNSCCs, and particularly laryngeal tumors, is of utmost importance, since most of the patients eligible for immunotherapy have been previously treated with neoadjuvant therapy, therefore possibly coming across these chemo/radio-induced PD-L1 fluctuations. Upregulation of PD-L1 following exposure to different kinds of chemoagents has already been described in other solid tumors such as breast and ovarian cancers [69,70,71]. Several studies aimed to assess the trend of PD-L1 expression in HNSCCs in cohorts of samples, including laryngeal cancers following chemo and radiotherapy, and they led to heterogenous results. The occasional discordance of such findings is easily explainable considering the different regimens of treatment, the specific systemic therapy associated with radiotherapy (e.g., cisplatin versus cetuximab), the scoring system chosen for the evaluation, and the antibody clone used for the immunohistochemical assessment of PD-L1. Moreover, it is hard to sharply separate the effects of radiotherapy and chemotherapy on PD-L1 expression, since these treatments are usually administered in conjunction in the neoadjuvant setting [72].

RT increases IFN type I secretion, which, albeit enhancing the activation of the antitumoral immune response, promotes the expression of PD-L1 on neoplastic cells, thus implying a concomitant suppression of immune activity. An in vivo study on mice has shown that administration of ionizing radiations on tumor microenvironment upregulates PD-L1 expression on tumor cells, DCs, and, less intensely, on macrophages [73]. Upregulation of PD-L1 has been demonstrated by Ock et al. both in vitro and in vivo on HNSCCs tumor cells exposed to cisplatin. Overall, they observed a treatment-induced variation of PD-L1 expression in 37.1% of cases, with a strong deviation in cisplatin-treated samples. Moreover, the most striking alteration was recorded in pretreatment negative samples that showed a positivization for PD-L1 in 69.2% of cases following cisplatin treatment. Interestingly, they also demonstrated an activation of the MEK pathway in association with PD-L1 upregulation, suggesting that regulation of MEK protein could exert a role in PD-L1 modulation [41]. Likewise, a study evaluating the expressions of PD-L1 on tumor and immune cells after TPF induction chemotherapy in a cohort of HNSCCs reported an overall significant increase in PD-L1 levels after treatment. In detail, using a cutoff of 5%, PD-L1 positivity was found to increase from 24% to 71% on immune cells and from 9.5% to 38% on tumor cells, before and after TPF induction chemotherapy, respectively [36]. Similarly, Karabajakian et al. focused on the comparison between PD-L1 expression in the primary tumor and in the recurrence of HNSCCs, including laryngeal cancers. Noteworthily, most of the negative samples in the primary diagnosis (75%) showed a positivization in the recurrence, and one can assume this modification to be attributable to the effects of treatments. In fact, almost all the patients received radiotherapy, alone or in combination with chemotherapy or cetuximab [68]. Another study on a cohort of patients with recurrent HNSCC treated with radiotherapy in conjunction with cisplatin demonstrated a treatment-induced increase of PD-L1 expression [42]. In this case, PD-L1 assessment was made separately, both on tumor cells and immune cells, and a significant variation of PD-L1 expression was noted especially in neoplastic cells. In particular, 31.8% of originally negative cases turned out to be PD-L1-positive on tumor cells in posttreatment recurrences [42]. Based on these findings, it could be postulated that radiation-induced upregulation of PD-L1 could favour neoplastic recurrence through the inhibition of T-cells functions induced by PD-1-PD-L1 axis in the tumor microenvironment [42]. However, this trend of increasing PD-L1 expression following radio- and chemotherapy in HNSCCs has not been confirmed in other studies. In a study comprising nine locally advanced laryngeal SCCs treated with chemoradiotherapy with curative purposes, no differences of PD-L1 levels were observed between preoperative and postoperative specimens. Most patients were treated with cisplatin or carboplatin, while a minority received cetuximab or mitomycin C [43]. A study by Ono et al. evaluating immune parameters in local recurrences of HNSCCs obtained similar findings. All patients had histories of radiotherapy and cisplatin-based chemotherapy, and a comparative evaluation between pre-CTRT and post-CTRT biopsy was made, highlighting no significant differences in PD-L1/PD-L2 expressions between the samples [37].

Regarding PD-L1 expression in metastases, the comparison between lymph node metastases and primary tumors treated with radiotherapy and surgery demonstrated a significative correlation in the PD-L1 expression pattern between primary tumors and metastases [74]. Another study including only SCCs of the oral cavity evaluated PD-L1 expression in lymph node metastases and recorded an increase trend of PD-L1 levels in lymph node metastases compared to primary tumors. It is reasonable to consider the lymphatic environment as possibly responsible for a selective pressure on tumor cells, which in turn are prone to develop immune escape with subsequent upregulation of PD-L1 [75]. Although not always being concordant, taken together these results suggest an overall increase of PD-L1 expression in laryngeal tumors following neoadjuvant therapies. This evidence provides a rationale for the combination of radio-chemotherapy with immune checkpoint inhibitors, since the latter could enforce the antitumoral effects of the former by blocking the immune response suppression pathway of the PD-L1-PD-1 axis, which is one of the main immunosuppressive effects of radiotherapy. On the other hand, augmentation of PDL1 after chemotherapy likely represents a mechanism of tumoral immune-mediated resistance to treatment, and its impairment could be crucial to unleashing a stronger therapeutic effect of chemoagents [36]. In light of this evidence, a re-evaluation of PD-L1 on posttreatment tumor samples regardless of the PD-L1 status of the primary tumors is strongly recommended, in order to allow access to immune checkpoint treatment in case of positivization [72]. Exploiting the beneficial effect of such mechanisms is fundamental because despite the implementation of multimodal treatments, 40–50% of patients with advanced disease undergo local or distant recurrences [6,7]. Nonetheless, studies about immune checkpoint inhibitors have demonstrated significant improvements in the outcomes of patients with locally advanced or metastatic HNSCCs, and PD-L1 expression by tumor and immune cells still accounts for the main predictive biomarker of good response [36]. Moreover, in laryngeal cancers, high levels of PD-L1 expression by immune cells are associated with better outcomes in terms of disease-free survival and overall survival. This apparent discrepancy can be explained by the fact that PD-L1 expression is increased by IFN type I, which is secreted by activated T-cells. Thus, high levels of PD-L1 mirror the presence of a good antigen-induced antitumor T-cell response in the TME [65,76]. However, a solid and broad study focusing on the effects of radiotherapy and chemotherapy on the PD-L1 status of laryngeal cancer is lacking, and it would be important to gain more insights about its possible implications for the outcomes of patients with advanced SCC undergoing immunotherapy in association with conventional therapies.

## 6. Conclusions

In summary, recurrent laryngeal SCC presents profound histopathologic differences, both from the primary tumor and, more importantly, according to the type of primary treatment. The modifications induced by RT in the tumor are more striking than those seen in postsurgical recurrences, and concern both the tumor cells and the microenvironment. They are strictly related to the tissue changes induced by RT (necrosis, fibrosis, modifications of the tumor vessels), and besides being a potential source of diagnostic problems, they subtend profound changes of the tumor immune microenvironment. Indeed, RT, CHT, and other systemic treatments can modify the TME of laryngeal SCC, exerting both immunosuppressive and immunostimulatory effects. Overall, the increases in immunoregulatory cells and immune checkpoints such as CTLA-4, TIM-3, PD-1, and PD-L1 induced by RT and CHT strongly support the use of ICI in recurrent/persistent laryngeal SCC. Future studies aiming to identify predictive factors of the response to ICI should consider such treatment-induced modifications.

## Figures and Tables

**Table 1:** Summary of the immune tumor microenvironment treatment-induced changes in head and neck squamous cell carcinoma.

Reference	Laryngeal Tumors (%)	Setting of the Study	Previous Treatment	Sample Type	TME Modifications and Essential Findings	Method for the Evaluation	Clinical Significance
[36]	38	Locally advanced primary tumor	Induction CT (TPF)	Resection specimen	Increase of CD8+ and Foxp3+ cell density; increase of CD8+/Foxp3+ ratio; increase of PDL-1 expression on TC and IC	IHC	Better prognosis and better response to ICIs
[37]	60	Recurrence	CT (cisplatin) + RT; RT	Resection specimen	Positive correlation between increase of CD8+ cell density and PD-L1 expression on TC	IHC	Better prognosis
[38]	11	Locally advanced primary tumor	Cetuximab	Tumor tissue processed with mechanical dissociation	Increase of T-reg Foxp3+ cell density	Flow Cytometry	Immune suppression Impairment of Cetuximab-induced immunity; association with ICIs reasonable
[39]	11	Locally advanced primary tumor	Cetuximab	Tumor tissue processed with mechanical dissociation	Increase of PD1+ CD8+ and of TIM3+ CD8+ cell density	Flow Cytometry	Immune suppression Impairment of Cetuximab-induced immunity; Association with ICIs reasonable
[40]	6	Recurrence	Induction CT; Cetuximab; RT	Not specified	PD-L1 upregulation; heterogeneity of PD-L1 expression in time	IHC	Importance of PD-L1 evaluation prior to ICIs treatment
[41]	Not provided	Locally advanced primary tumor	Induction CT or CT + RT	Not provided	PD-L1 upregulation	IHC	Importance of PD-L1 evaluation prior to ICIs treatment
[42]	20.5	Recurrence	CT + RT	Resection specimen or biopsy	Heterogeneity of PD-L1 expression in time	IHC	Importance of PD-L1 evaluation prior to ICIs treatment
[43]	13.4	Locally advanced primary tumor	Definitive CT + RT	Biopsy	No modification of PD-L1 and PD-1 expression; CD27 downregulation on IC	IHC	Further investigations needed

Abbreviations: CT: chemotherapy; IC: immune cells; ICIs: immune checkpoint inhibitors; IHC: immunohistochemistry; RT: radiotherapy; TC: tumor cells; TPF: docetaxel, platinum, and fluorouracil.

## Data Availability

Not applicable.
